# Animal and Cellular Studies Demonstrate Some of the Beneficial Impacts of Herring Milt Hydrolysates on Obesity-Induced Glucose Intolerance and Inflammation

**DOI:** 10.3390/nu12113235

**Published:** 2020-10-22

**Authors:** Rachel Durand, Adia Ouellette, Vanessa P. Houde, Frédéric Guénard, Thibaut V. Varin, Bruno Marcotte, Geneviève Pilon, Erwann Fraboulet, Marie-Claude Vohl, André Marette, Laurent Bazinet

**Affiliations:** 1 Department of food Sciences and Laboratory of Food Processing and Electromembrane Process (LTAPEM), Université Laval, Québec, QC G1V 0A6, Canada; rach.dura@laposte.net; 2 Institute of Nutrition and Functional Foods (INAF), Université Laval, Québec, QC G1V 0A6, Canada; adia.ouellette.1@ulaval.ca (A.O.); vanessa.houde@fmd.ulaval.ca (V.P.H.); Frederic.Guenard@fmed.ulaval.ca (F.G.); thibaut.varin.1@ulaval.ca (T.V.V.); Bruno.Marcotte@criucpq.ulaval.ca (B.M.); Genevieve.Pilon@criucpq.ulaval.ca (G.P.); Marie-Claude.Vohl@fsaa.ulaval.ca (M.-C.V.); Andre.Marette@criucpq.ulaval.ca (A.M.); 3 Québec Heart and Lung Institute, Department of medicine, Université Laval, QC G1V 4G5 Québec, Canada; 4 School of Nutrition, Université Laval, Québec, QC G1V 0A6, Canada; 5 Ocean NutraSciences Inc., Matane, QC G4W 3M6, Canada; Erwann_Fraboulet@uqar.ca

**Keywords:** herring milt hydrolysate, bioactive peptides, polyunsaturated fatty acids, obesity, glucose tolerance, microbiota, *Lactobacillus*

## Abstract

The search for bioactive compounds from enzymatic hydrolysates has increased in the last few decades. Fish by-products have been shown to be rich in these valuable molecules; for instance, herring milt is a complex matrix composed of lipids, nucleotides, minerals, and proteins. However, limited information is available on the potential health benefits of this by-product. In this context, three industrial products containing herring milt hydrolysate (HMH) were tested in both animal and cellular models to measure their effects on obesity-related metabolic disorders. Male C57Bl/6J mice were fed either a control chow diet or a high-fat high-sucrose (HFHS) diet for 8 weeks and received either the vehicle (water) or one of the three HMH products (HMH1, HMH2, and HMH3) at a dose of 208.8 mg/kg (representing 1 g/day for a human) by daily oral gavage. The impact of HMH treatments on insulin and glucose tolerance, lipid homeostasis, liver gene expression, and the gut microbiota profile was studied. In parallel, the effects of HMH on glucose uptake and inflammation were studied in L6 myocytes and J774 macrophages, respectively. In vivo, daily treatment with HMH2 and HMH3 improved early time point glycemia during the oral glucose tolerance test (OGTT) induced by the HFHS diet, without changes in weight gain and insulin secretion. Interestingly, we also observed that HMH2 consumption partially prevented a lower abundance of *Lactobacillus* species in the gut microbiota of HFHS diet-fed animals. In addition to this, modulations of gene expression in the liver, such as the upregulation of sucrose nonfermenting AMPK-related kinase (SNARK), were reported for the first time in mice treated with HMH products. While HMH2 and HMH3 inhibited inducible nitric oxide synthase (iNOS) induction in J774 macrophages, glucose uptake was not modified in L6 muscle cells. These results indicate that milt herring hydrolysates reduce some metabolic and inflammatory alterations in cellular and animal models, suggesting a possible novel marine ingredient to help fight against obesity-related immunometabolic disorders.

## 1. Introduction

The Western diet, which is typically rich in fat and sugar, contributes to obesity development worldwide and in turn contributes to the development of metabolic disorders, including insulin resistance and dyslipidemia. Consequently, the risk of type 2 diabetes (T2D) and cardiovascular disease (CVD) is then increased [1,2]. To prevent the development of these disorders, prevention programs emphasize lifestyle changes such as diet modification [1]. The consumption of fish has been reported to decrease the risk of T2D and CVD in epidemiological studies [3,4,5]. For decades, polyunsaturated fatty acids (PUFA), particularly omega-3 (ω-3), found in fatty fish, have been associated with these health benefits. Indeed, the consumption of fish oil has been linked to an improvement of dyslipidemia and insulin resistance in patients with metabolic disorders [6,7,8]. It is worth noting that other bioactive compounds in fish, such as essential amino acids (AA) or peptides, have also been associated with an improvement of glucose and lipid homeostasis in animal and human studies [9,10,11,12,13,14,15,16] Liaset et al. [17] reported that a high concentration of lysine and taurine in a saithe hydrolysate promoted the secretion of hepatic bile acids in rats, thus reducing visceral adipose tissue and inducing the expression of genes involved in fatty acid oxidation. Our group has also previously demonstrated that the replacement of 50% of the protein source of a high-fat diet by a salmon protein hydrolysate improved the glucose tolerance and decreased dyslipidemia, as well as inflammation, in the adipose tissue of in-house colony atherosclerotic LDLR-/-/ApoB(100/100) mice [18]. Interestingly, in overweight subjects, the consumption of a diet composed of 58–68% of daily dietary proteins from cod was found to improve the insulin sensitivity compared to protein sources from lean beef, pork, veal, eggs, milk, and dairy products [16]. Although these types of studies are very important as a clinical proof of concept, keeping up this type of diet on a regular basis can be difficult, making fish-based supplements a likely solution. In the last 15 years, a considerable number of studies have reported an association between gut microbiota modification and metabolic disorders, such as obesity, type 2 diabetes, and fatty liver diseases. Although studies reporting the impact of PUFA on gut microbiota are accumulating, very few studies have looked at the role of fish proteins for their capacity to influence this now well-recognized and important element of metabolic health [19]. Recently, Hosomi et al. reported that rats consuming a diet containing only Alaskan pollock instead of casein as a protein source exhibited an improvement in their glucose metabolism, which was associated with a change in the composition of their gut microbiota [20]. However, to the best of our knowledge, the impact of a realistic daily dose of fish products taken in the form of a supplement has not yet been studied in this context.

The fishing industry produces large amounts of by-products (heads, viscera, bones, skin, and milt/roe), of which an average of 50% are discarded after processing [21]. These by-products contain valuable molecules that could be used for human nutrition and health purposes. Regarding herring processing, the milt could represent a promising co-product rich in proteins and lipids. Indeed, we previously identified two anti-inflammatory peptides from a herring milt hydrolysate obtained from separation by electrodialysis with an ultrafiltration membrane [22]. Moreover, Wang et al. observed an improvement of insulin resistance in obese mice fed a high-fat diet in which a large proportion of the protein source (70%) was from herring milt hydrolysate [23]. However, in order to develop a nutraceutical product, an achievable and effective dose for daily human consumption must be determined.

The aim of this study was to evaluate the impact of low doses of different herring milt hydrolysates, which contain a mix of peptides, lipids, and astaxanthin, on the development of obesity-related metabolic disorders in mice fed a high-fat high-sucrose (HFHS) diet and in cellular models.

## 2. Materials and Methods

### 2.1. Herring Milt Hydrolysate Extracts

Three industrial products containing herring milt hydrolysate (HMH1, HMH2, and HMH3) were provided by Ocean NutraSciences (Matane, QC, Canada). Briefly, herring milt was hydrolyzed with a mix of enzymes (confidential) and separated by ultrafiltration membranes. HMH1 (commercial name PG-1) was made of the permeate, HMH2 (commercial name Sementis) of the retentate, and HMH3 (no marketed product) of the retentate supplemented with astaxanthin. The composition of each product is listed in Table 1.

### 2.2. Animals and Dietary Treatment

Six-week-old C57Bl/6J male mice from The Jackson Laboratory (Bar Harbor, Maine, USA) were housed individually in ventilated cages and acclimatized for 12 days under a 12 h daylight cycle and constant temperature. Chow diet (Teklad 2018, Harlan) and water were provided ad libitum. Following the acclimatization period, the mice were either kept on the chow diet or fed an HFHS diet containing 65% lipids, 15% protein, and 20% carbohydrates. Experiments were carried out in four batches, with each batch consisting of three mice for each of the five treatments, thus resulting in a total of 60 mice. Mice were randomly assigned to the different treatments (*n* = 12 per treatment) as follows: Three groups were put on the HFHS diet (HMH1, HMH2, and HMH3) and received herring milt hydrolysate at a dose of 208.8 mg/kg (equivalent to a 1 g/day consumption for a human) by daily oral gavage. This calculation is based on the FDA equation which takes into consideration the different metabolic rates between species [24]. Human equivalent dose (HED) = mouse dose mg/kg x (Km mouse/Km human), where the KM for a mouse is 3 and 37 for a 60 kg human.

The two remaining groups (chow and HFHS) received the vehicle (water) by daily oral gavage. Fresh feces were collected from each mouse before the start and at the end of the protocol by putting each mouse in an alcohol-cleaned plexiglass cage, in which the feces were quickly collected in sterile tubes using sterilized pliers and rapidly frozen in liquid nitrogen and then stored at −80 °C. Body weight gain and food intake were assessed twice a week. After 8 weeks of treatment, mice were anesthetized by isoflurane inhalation and blood samples were collected by cardiac puncture from which the plasma was subsequently separated. Tissues were collected, weighed, and stored at −80 °C until further analysis. All procedures met the guidelines for the care and use of laboratory animals and were approved by the Laval University Animal Ethics Committee: 2017134-1.

### 2.3. Insulin Tolerance Test

After 6 weeks of treatment, the mice were fasted for 6 h in preparation for the insulin tolerance test (ITT), which was performed by an intraperitoneal injection of insulin (0.65 U/kg of body weight). Blood was drawn from the caudal vein and glycemia was measured with a OneTouch Verio Flex glucometer (LifeScan Canada, Burnaby, BC, Canada) before (0 min) and after (5, 10, 20, 30, and 60 min) the insulin injection.

### 2.4. Oral Glucose Tolerance Test

Three days before the sacrifice, mice were fasted for 12 h and an oral glucose tolerance test (OGTT) was performed. Briefly, 1 g of dextrose/kg of body weight was given orally to each mouse and blood was drawn from the caudal vein and processed by a OneTouch Verio Flex glucometer (LifeScan Canada), in order to measure glycemia before (0 min) and after (15, 30, 60, and 120 min) the oral administration of glucose. Blood samples (30 µL) were also collected at each time point for insulin determination.

### 2.5. Biochemical Analysis

The liver triglycerides (TG) and cholesterol were processed by chloroform-methanol extraction based on a modified Folch method [25], and measured by an enzymatic reaction with colorimetric assay kits (ThermoFisher Scientific, Mississauga, ON, Canada and Randox Laboratories, Crumlin, UK). The plasma insulin was analysed using an ultrasensitive ELISA kit (Alpco, Salem, NH, USA), in accordance with the manufacturer′s instructions.

### 2.6. Fecal Sample Processing and 16S rRNA Gene-Based Sequencing

At weeks 0 and 8, fresh fecal samples were collected and stored at −80 °C. Bacterial DNA was extracted from the collected samples and purified using a ZymoBIOMICS^®^ DNA Kit (Zymoresearch, Ivrine, CA, USA), in accordance with the manufacturer’s instructions. The V3-V4 region of the 16S rRNA gene of the DNA extracts was then amplified using 341F (5′-CCTACGGGNGGCWGCAG-3′) and 805R (5′-GACTACHVGGGTATCTAATCC-3′) primers, which are adapted to incorporate the transposon-based Illumina Nextera adapters (Illumina, San Diego, CA, USA) and a sample barcode sequence, both of which allow for multiplexed paired-end sequencing. The amplification mix contained 1× Q5 buffer (NEB), 1× Q5 Enhancer (NEB), 200 μM dNTP (VWR International, Mississauga, Ontario, Canada), 0.2 μM of forward and reverse primers (Integrated DNA Technologies, Coralville, Iowa USA), 1 U of Q5 (NEB), and 1 μL of template DNA in a 50 μL reaction. The cycling condition was as follows: Denaturation (30 s at 98 °C), followed by a first set of 15 cycles (98 °C for 10 s, 55 °C for 30 s, and 72 °C for 30 s), a second set of 15 cycles (98 °C for 10 s, 65 °C for 30 s, and 72 °C for 30 s), and a final elongation period (2 min at 72 °C). The construction and sequencing of 16S rRNA gene-based libraries were performed as previously described [26]. All raw sequences were deposited in the public European Nucleotide Archive server under accession number PRJEB36103 (https://www.ebi.ac.uk/ena/data/view/PRJEB36103).

### 2.7. Gut Microbiota Analysis

Forward and reverse primers were removed from raw paired-end reads using Cutadapt (v1.14; Martin et al., 2011) [27]. Sequences were then demultiplexed, denoised, dereplicated, and merged with the DADA2 package in the R software [28]. The resulting amplicon sequence variants (ASVs) table was assigned taxonomy using the Ribosomal Database Project RDP classifier algorithm (v2.2; Wang et al., 2007), trained against the SILVA database 132 (Quast et al., 2013) [29,30]. Samples were rarefied to an even sampling depth of 10,456 sequences per sample. 

### 2.8. Bacterial Quantification by Real-Time qPCR

The presence of *Lactobacillus* sp. in the mice feces was assessed by qPCR, as previously described by Anhê et al. (2018) [31]. The relative quantity of *Lactobacillus* sp. was calculated based on the Ct obtained using a standard curve. The sequence of the *Lactobacillus* sp. primers used was: Forward: 5′-AGCAGTAGGGAATCTTCCA-3′, and reverse: 5′-CGCCACTGGTGTTCYTCCATATA-3′ [32].

### 2.9. Liver RNA Extraction

Total RNA was extracted with Trizol (ThermoFisher Scientific) from 20–30 mg of freeze-powdered liver tissue collected at sacrifice (week 8). This was followed by purification with the GeneJET RNA purification kit from ThermoFisher Scientific, in accordance with the manufacturer’s instructions. The RNA was then cleaned with the GeneJET RNA Cleanup and Concentration Micro Kit (ThermoFisher Scientific) prior to quantification with a spectrophotometer.

### 2.10. Liver RNA-Sequencing

Equal amounts of RNA from three mice under the same treatment were pooled (three samples from the same batch per pool), thus resulting in four pools for each treatment. An amount of 4000 ng/pool was sent for RNAseq analysis at McGill University and the Génome Québec Innovation Centre. The quality of the RNA samples was evaluated with an Agilent Bioanalyzer 2100. All samples displayed an RNA integrity number (RIN) score > 7 and were retained for further analyses. RNA samples were converted to cDNA with the Illumina RNA seq kit (NEBNext_dual_i7_A10-NEBNext_dual_i5_A10; rRNA-depleted stranded (HMR)) for sequence library preparation based on the manufacturer’s protocol. Final libraries were sequenced on an Illumina NovaSeq6000 S2 sequencer using paired-end, 100 bp reads.

Raw reads were trimmed for the length (*n* = 50), quality (phred33 score ≥ 30), and adaptor sequence using trim_galore v0.4.4, a wrapper tool around Cutadapt (v1.9.1), and FastQC (v0.11.8). Reads were aligned against the GRCm38/mm10 assembly of the mouse transcriptome (RefSeq mRNA) using the kallisto quant command (v0.44.0) with the default parameters and 100 bootstraps. Raw read counts and normalized read counts (in transcripts per million, TPM) were thus obtained [33]. Estimated counts were obtained and differential transcript expression was determined using edgeR v3.24.3 on R v3.5.1, taking into account the batch number as a confounding factor [34].

### 2.11. Cellular Bioactivity

#### 2.11.1. Glucose Uptake

The glucose uptake was measured with L6 skeletal muscle cells grown in α-MEM media supplemented with 10% (*v/v*) fetal bovine serum (FBS). The cells were differentiated into myotubes in α-MEM media supplemented with 2% (*v/v*) FBS in an atmosphere of 5% CO_2_ at 37 °C, as previously described [35]. L6 cells were serum-deprived for 5 h prior to the experiments and 100 nM of insulin was used to stimulate the cells during the last 45 min of deprivation. Cells were also treated with 1 µg/mL, 1 ng/mL, and 100 pg/mL of each herring milt hydrolysate extract for the last 75 min of deprivation. The glucose uptake assay was performed as described previously [36].

#### 2.11.2. Inflammation

The potential anti-inflammation activity of each product was tested in J774 macrophages plated at 15 × 10^6^ cells in RPMI media supplemented with 10% (*v/v*) bovine growth serum (BGS, HyClone, ThermoFisher Scientific, Waltham, MA, USA) and 1% (*v/v*) penicillin. Each product was added at a final concentration of 1 µg/mL, 1 ng/mL, and 100 pg/mL and incubated for 24 h. To induce inflammation, lipopolysaccharide (LPS) was added at a final concentration of 2.5 ng/mL for the last 18 h of the incubation period and the nitrite concentration was measured in the media, as previously described, by the Griess method [36].

#### 2.11.3. Statistical Analysis

To analyse the effect of the HFHS diet on mice, Student’s *t* tests were conducted for the chow and HFHS diet. Differences in HMH diets and HFHS control diets were assessed by an ANOVA with a post-hoc Dunnett test. Time points within the different groups for the OGTT and ITT were compared using a two-way repeated measure ANOVA with a post-hoc Student–Newman–Keuls test. All statistical analyses for the in vivo protocol were assessed with SigmaPlot 12.0.

All statistical tests for the cell culture protocol were carried out in SAS software version 9.3 (SAS Institute Inc., Cary, NC, USA). One-way ANOVA tests were completed to verify the differences in glucose uptake values and inflammation markers with a post-hoc Dunnett test to identify differences between groups. The normality and residue homogeneity were controlled by the Shapiro test and Levene test, respectively.

Regarding gut microbiota data, differences in beta diversity were visualized with principal coordinate analysis (PCoA) based on the weighted UniFrac distance, as described previously [37]. The detection of differentially abundant taxa or pathways between groups was performed with the metagenomics biomarker discovery tool for linear discriminant analysis effect size [38].

Differential transcript expression in the liver was conducted using edgeR (v3.24.3) in R and based on the experimental design and planned comparisons. The HFHS diet was first compared to the chow diet to reveal changes in the transcript expression following the HFHS diet. In addition, each HFHS diet supplemented with an HMH product (either HMH1, HMH2, or HMH3) was compared to the HFHS diet. False discovery rate-corrected *p*-values (Benjamini–Hochberg) were calculated to account for multiple comparisons.

## 3. Results

The impact of the HFHS diet on the physiological parameters is presented in Table 2. As expected, the HFHS diet increased the total weight gain and all fat depots in mice compared to the chow-fed group. However, none of the HMH treatments had an impact on the physiological characteristics, except for the increase of the total energy intake in HMH1-treated animals. Since obesity is closely linked to the development of fatty liver diseases, we then investigated the impact of HMH treatments on liver steatosis. The HFHS diet did not increase the liver weight in comparison to the chow diet (Table 2), but did increase the hepatic TG and cholesterol content. However, HMH treatments did not prevent lipid accumulation in the organ.

**Figure 1:**
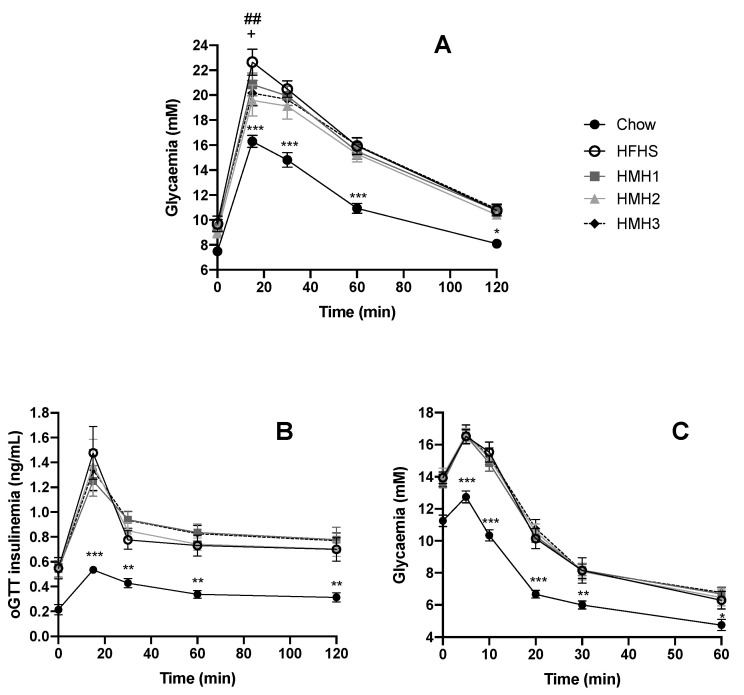
Herring milt hydrolysate (HMH2 and HMH3) improved early time point glycemia during the oral glucose tolerance test (OGTT) (*n* = 11−12). (**A**) Glycemic curve during the OGTT for HMH1, HMH2, and HMH3 treatment at a dose of 208.8 mg/kg; (**B**) insulin production during the OGTT for HMH1, HMH2, and HMH3 treatment; (**C**) glycemic curve during the insulin tolerance test (ITT) for HMH1, HMH2, and HMH3. Time points within the different groups were compared using a two-way repeated measure analysis of variance (ANOVA) with a post-hoc Student–Newman–Keuls test. * *p* < 0.05 chow vs. HFHS, ** *p* < 0.01 chow vs. HFHS, *** *p* < 0.001 chow vs. HFHS, ^##^ *p* < 0.01 HFHS vs. HMH2, and ^+^ *p* < 0.05 HFHS vs. HMH3. HFHS: high-fat high-sucrose,.

We next investigated the impact of HMH treatments on glucose and insulin tolerance (Figure 1). As expected, the chow-fed animals showed a significantly lower fasting glycemia level and glycemic response during both the ITT and OGTT compared to the HFHS control group. Interestingly, during the OGTT, animals treated with HMH2 and HMH3 exhibited a decrease in glycemia in comparison to the HFHS vehicle-treated animals 15 min post glucose challenge (Figure 1A). However, glucose values were comparable between HFHS animals at later time points, and the total area under the curve (AUCs) was thus not different between HFHS groups (data not shown). Glucose-stimulated insulin responses during the OGTT (Figure 1B) and the glucose response to the ITT (Figure 1C) did not reveal any effect on insulin sensitivity in HMH-treated mice, suggesting that the lower glucose peak observed during the OGTT may be independent of the improved insulin action on glucose disposal.

### 3.1. Liver Transcript Expression Analysis

Using RNAseq analysis, we next assessed the potential effect of HMH treatments on global transcript expression levels in the liver. As expected, HFHS vehicle-treated mice displayed numerous changes in transcript expression levels in comparison to the healthy chow diet-fed mice (Table S1). Briefly, among the 15,334 transcripts detected in the liver, 1040 were found to be underexpressed and 954 were overexpressed. Pathway analysis conducted from the list of differentially expressed transcripts following the HFHS vehicle-treated diet revealed the enrichment of pathways related to insulin resistance, carbohydrate metabolism (glycolysis/gluconeogenesis, pentose and glucuronate interconversions, ascorbate and aldarate metabolism, and pyruvate metabolism), lipid metabolism (fatty acid degradation and arachidonic acid metabolism), and amino acid metabolism (lysine degradation and tryptophan metabolism) to be among the top 20 enriched pathways (Table S1). In contrast, a low number of transcripts were differentially expressed in HMH-treated groups compared to the HFHS group treated with the vehicle. HMH2 supplementation was linked to the differential expression of five transcripts: Laminin subunit beta-3 (Lamb3), interferon regulatory factor 1 (Irf1), and Nuak2 or sucrose nonfermenting AMPK-related kinase (SNARK) were over regulated, while pleckstrin homology like domain family A member 1 (Phlda1) and forkhead box Q1 (Foxq1) were down regulated following correction for multiple testing (FDR-adjusted *p* < 0.05-value; Table S1). The transcript encoding for the laminin subunit beta-3 was also found to be over regulated under supplementation with HMH1 in comparison to the HFHS diet control. No modulation was reported for the HMH3 treatment (Table S1).

### 3.2. Effects of HMH Treatment on the Gut Microbiota

**Figure 2:**
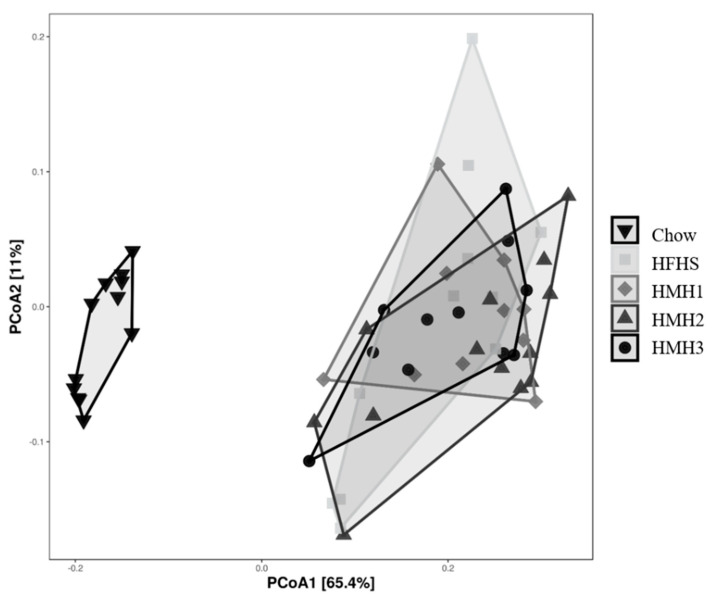
None of the HMH treatments impacted the global structure (Beta-diversity) of the gut microbiota after 8 weeks, as reflected by the principal coordinates analysis (PcoA) on the weighted UniFrac distance matrix. (*n* = 11−12).

**Figure 3:**
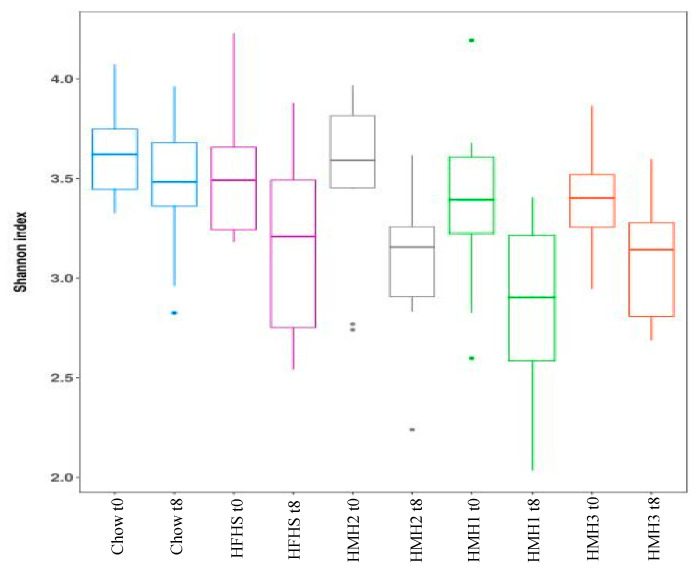
None of the treatments impacted the Alpha-diversity of gut microbiota before and after the different treatments, as measured with the Shannon index. (*n* = 11–12).

Fecal samples were collected before and at the end of the different treatments to investigate the impact of HMH products on the gut microbiota at the genus level using 16S rRNA gene sequencing. As expected, the gut microbiota composition clustered differently for the chow and all HFHS-treated groups after 8 weeks of treatment. Indeed, the PcoA on the weighted UniFrac distance (Figure 2) exhibited a separation into two clusters, depending on the diet. The gut microbiota of the HMH treated mice, however, were not clustered differently to the control HFHS-fed mice. Alpha-diversity was further estimated by measuring the Shannon index for each group (Figure 3). After 8 weeks of treatment, a decrease of the diversity index was observed in all the HFHS-treated groups, while the Shannon index for the chow diet remained unchanged during experimentation.

**Figure 4:**
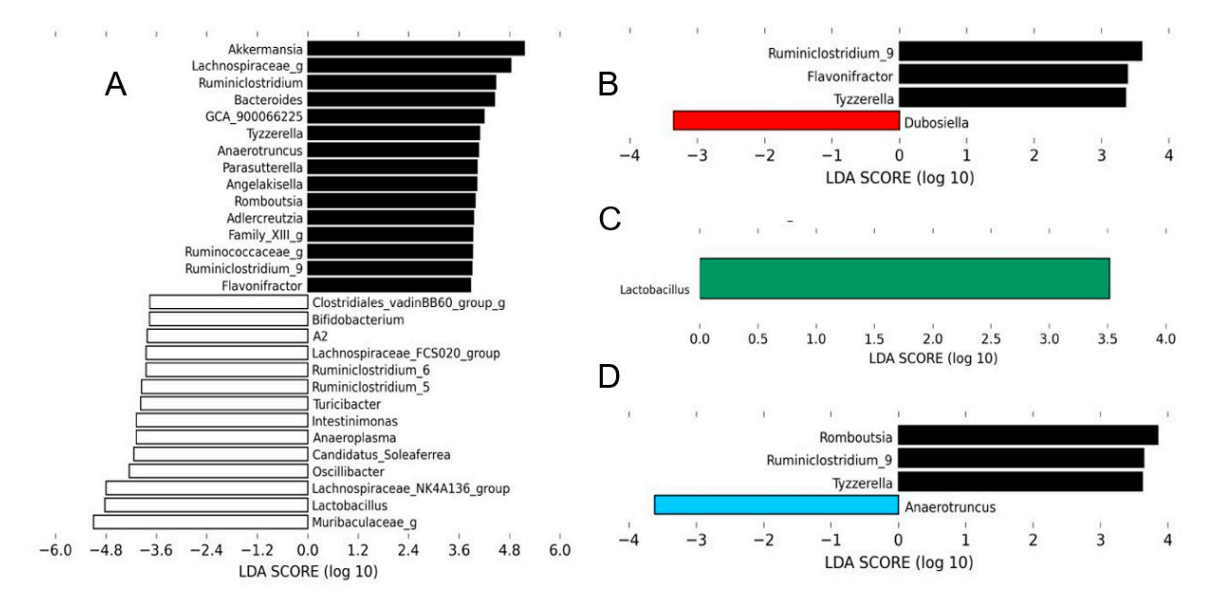
Each HMH treatment induced changes in the gut microbiota at the genus level. The linear discriminant analysis (LDA) effect size was calculated in order to explore the taxa within genus levels that more strongly discriminated between the gut microbiota of mice fed with (**A**) chow (white) and high-fat high-sucrose (HFHS), (**B**) HFHS and HMH1 (red), (**C**) HFHS and HMH2 (green), and (**D**) HFHS and HMH3 (blue). The statistical significance of differentially abundant bacteria between the two distinct biological conditions was measured using linear discriminant analysis effect size (LEfSe). Black bars represent HFHS.

The LEfSe analysis of the gut microbiota revealed that mice treated with HMH1 were discriminated from those of untreated HFHS mice, as shown by an increased abundance of *Dubosiella* and a reduction of the *Ruminoclostridum, Flavonifractor*, and *Tyzzeralla* order (Figure 4B). Interestingly, the HMH2 treatment allowed for the expansion of the *Lactobacillus* population in comparison to the HFHS control, which was in turn decreased in comparison to the gut microbiota of chow-fed mice (Figure 4A,C). This over representation of *Lactobacillus* in the HMH2-treated mice was further confirmed by PCR analysis (Figure S2). The gut microbiota profile of HMH3-treated mice had a higher proportion of *Anaerotruncus* and a lower abundance of *Ruminoclostridum, Tyzzerella*, and *Romboustia* when compared to HFHS control mice.

### 3.3. Glucose Uptake and Inflammation

**Figure 5:**
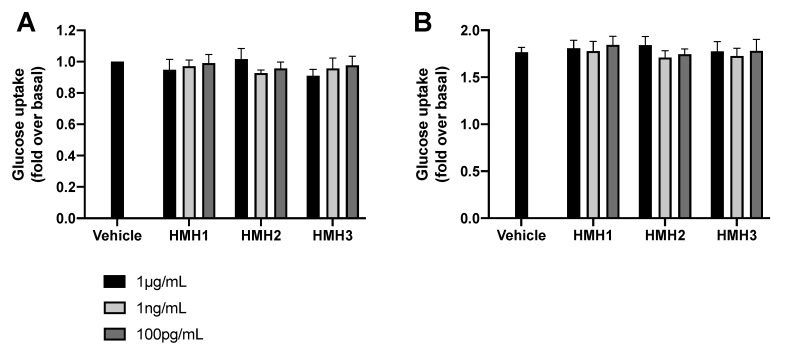
None of the HMH treatments impacted the glucose uptake in L6 myocytes. (**A**) Glucose uptake without insulin stimulation and (**B**) glucose uptake with insulin stimulation in L6 cells treated with HMHs at three concentrations (1 µg/mL, 1 ng/mL, and 100 pg/mL) (*n* = 6, mean ± SEM). SEM: standard error of the mean.

Tests were carried out on cultured cells to identify the bioactivity of the HMHs, which could partially explain the effects observed in mice. Glucose uptake was determined with and without insulin stimulation in L6 muscle cells (Figure 5). These conditions were previously used to reveal the glucoregulatory effects of other hydrolysates and peptidic fractions [18]. None of the three HMH products were found to modulate glucose uptake and insulin action in the myocytes at any of the concentrations tested.

**Figure 6:**
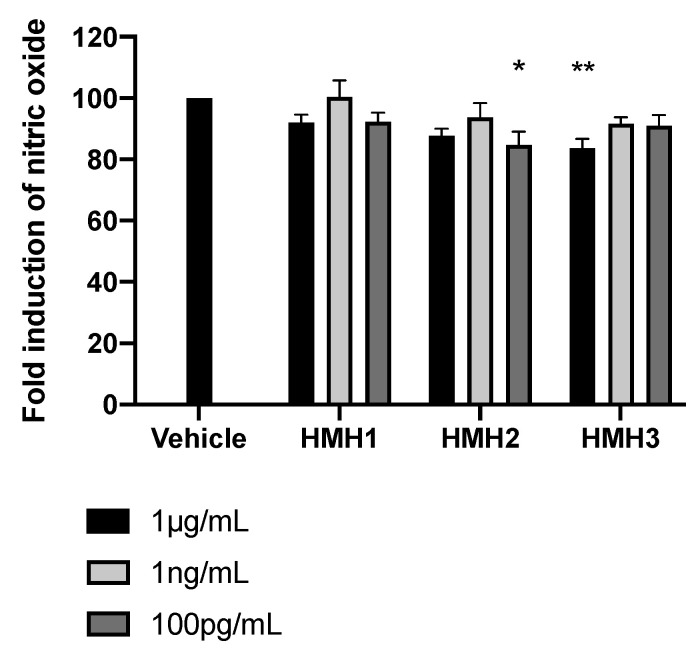
HMH3 reduced nitrite production in LPS-induced J774 mice macrophages. Nitrite production in culture media after inflammation induction by LPS in mice treated with HMHs at three concentrations (1 µg/mL, 1 ng/mL, and 100 pg/mL), *n* = 6, means ± SEM, one-way ANOVA post hoc Dunnett test, * *p* < 0.05, and ** *p* < 0.01.

The anti-inflammatory bioactivity of the HMH products was then investigated by verifying nitrite production in LPS-stimulated J774 macrophages treated with HMH2 and HMH3. This type of analysis reflects the activity of iNOS, which is a well-known pro-inflammatory mediator of insulin resistance [36]. Although modest, significant and reproductible iNOS inhibition was observed in HMH2- and HMH3-treated cells at two concentrations, depending on the product, e.g., at 100 pg/mL for HMH2 (*p* < 0.05) and at 1 µg/mL for HMH3 (*p* < 0.01) (Figure 6).

## 4. Discussion

The aim of this study was to evaluate the effect of three different treatments with herring milt hydrolysate products on the development of obesity-related metabolic disorders in mice fed a HFHS diet. Although several studies have shown the bioactive effects brought on by the consumption of different fish protein hydrolysates, very few have studied the effects of herring milt on obesity-related metabolic disorders [23,39,40]. In the present study, daily treatment with HMH2 and HMH3 products was found to reduce early glycemia excursion in response to an OGTT in obese mice after 8 weeks of treatment. This is in accordance with previous work by Wang et al., who also reported an improvement of glucose homeostasis in mice fed a high-fat diet in which 70% of the casein was replaced by herring milt hydrolysate [23]. The authors observed a decrease of the fasting blood glucose level and glycemic curves during the OGTT after 5 and 8 weeks of treatment with their modified diet.

Obesity-associated glucose intolerance development is known to involve low-grade inflammation processes. Interestingly, Bjorndal et al. reported the trend of a reduction in the plasma level of cytokines (IL-5, IL-1β, IL-2, and CSF2) in hTNFα mice fed a high-fat diet supplemented with 15% herring milt [39]. Aiming to better understand the mechanism behind the improvement of the glycemic response observed here with HMH2 and HMH3 treatments, we investigated the direct anti-inflammatory potential of these hydrolysates for J774 macrophages, in order to observe the effects on iNOS induction. The reduction of nearly 20% of nitrite production in cells treated with both products could indicate the involvement of a direct anti-inflammatory process in the improvement of glucose metabolism reported.

On the other hand, although the present study highlights similar bioactivities of the herring milt to the studies of Wang et al. and Bjorndal et al., some differences can also be noted [23,39]. Indeed, whereas the daily administration of HMH products did not impact the hepatic cholesterol and triglycerides (TG) concentrations in mice fed an HFHS diet, Bjorndal et al. reported a decrease in plasma TG, but an increase of the TC and TG concentrations in the liver in hTNFα mice fed a high-fat diet supplemented with 15% herring milt. Even though the high cholesterol content in the herring milt diet may have aggravated the initial disruption of hepatic cholesterol metabolism in the transgenic hTNFα mice, the complete deterioration of hepatic lipid accumulation by the herring milt in transgenic hTNFα mice has not been explained by the authors [39].

Although HMH2 and HMH3 reduced early time point glucose excursion during the OGTT, they did not reverse insulin resistance in obese mice (no changes of insulin production during OGTT and glycemia during the ITT). Conversely, Wang et al. reported a decrease of the fasting serum insulin, HOMA-IR, and a protective effect on pancreatic β-cells in mice fed a high-fat diet in which 70% of the casein content was substituted for herring milt hydrolysate, highlighting the improvement in insulin sensitivity [23]. Moreover, the authors also revealed a decrease in body weight in mice fed this modified diet, explaining the reduction of fasting blood glucose. Therefore, it was concluded that the observed benefits of the herring milt hydrolysate directly resulted from weight reduction or one of the benefits associated with weight reduction, as well as independent improvements due to specific bioactive compounds found in the treatment [23]. In the present study, however, the improvement of glycemic excursion in response to the OGTT in HMH2- and HMH3-treated mice could not be explained by a weight reduction.

In order to develop a nutraceutical product, an achievable and effective dose for daily human consumption must be demonstrated. Realistically, the replacement of 70% of the protein content with herring milt hydrolysate, as was reported in the study by Wang et al., appears difficult to apply clinically [23]. Furthermore, the authors reported moderate effects on obesity-related metabolic disorders in obese mice following the replacement of 35% of the protein content with herring milt hydrolysate and no effects were observed following a 15% replacement. In the present study, a dose of 208.8 mg/kg, corresponding to a 1 g/day consumption for a human, showed beneficial effects in obese mice. The efficiency of HMH2 and HMH3 may be explained by their particular composition and most of all, by the concentration of their bioactive compounds by ultrafiltration separation. Indeed, the separation process used in the production of bioactive hydrolysates defines peptide populations by their weight and promotes bioactivity [41]. In accordance with this, Roblet et al. previously reported an improved glucose uptake in L6 myocytes, which was induced by a soy retentate hydrolysate treatment separated by an ultrafiltration membrane (10 kDa) [42]. HMH2 and HMH3 are the herring milt hydrolysate retentate after their separation by an ultrafiltration membrane, while HMH1 is the permeate. Hence, HMH1 is mostly composed of small-weight peptides and free amino acids, while HMH2 and HMH3 are composed of higher-weight peptides and lipids [22,43]. These differences in composition may explain the higher efficiencies of HMH2 and HMH3 treatments. Indeed, in a previous study, the separation of HMH1 by electrodialysis with an ultrafiltration membrane (EDUF) led to the identification of two new anti-inflammatory peptides: IVPAS and FDKPVSPLL [22]. However, in the present study, HMH1 was reported to have no anti-inflammatory effect. This could be explained by the presence of inhibitory molecules and/or the use of an insufficient dose for the decrease of inflammation in cellular models. Moreover, a previous study has shown that HMH2 separation by EDUF improved the glucose uptake in cultured cells by the concentration of bioactive compounds and the removal of inhibitors [43]. The interaction between peptides and PUFA found in these hydrolysates may also improve this bioactivity [43]. Ouellet et al. highlighted that PUFA consumed in the form of fish are more available than those added to the diet by showing that the interaction between cod protein and PUFA allowed an improvement of insulin sensitivity in insulin-resistant individuals [16]. In the present study, HMH2 and HMH3 (composed of peptides, lipids, and astaxanthin in HMH3) improved glucose metabolism in obese mice, which could be the result of a synergetic relation between these different compounds. On the other hand, no modulations in glucose uptake in L6 muscle cells were observed following treatment with HMH2 and HMH3, suggesting that in vivo glucose metabolism improvement might not be due to a direct effect on the glucose transport in muscle, but to an indirect effect, such as the inhibition of inflammation. Indeed, HMH2 and HMH3 inhibited iNOS induction in macrophages known to contribute to the development of insulin resistance [44]. The production of TNF-α and IL-6 cytokines by macrophages leads to increased serine phosphorylation of insulin receptor substrate-1 (IRS-1), resulting in insulin resistance in the muscle and liver [45].

Moreover, the disruption of iNOS improves glucose metabolism by the protection of phosphatidylinositol 3-kinase and Akt activation [45]. Furthermore, it has been reported that fish oil prevented insulin resistance in the liver and muscle in rodents in a peroxisome proliferator–activated receptor (PPARα)-dependent manner due to natural PPARα ligands docosahexaenoic acid(DHA) and eicosapentaenoic acid (EPA) [46,47]. Finally, HMH3 is also composed of astaxanthin (Table 1). This carotenoid is well-known for its antioxidant activity, but some studies have also reported a beneficial effect on inflammation [48,49,50]. Lee et al. (2003) demonstrated an inhibition of nitric oxide (NO) production by astaxanthin with an half maximal inhibitory concentration (IC_50_) of 5 µM in macrophages stimulated with LPS [49]. Consequently, the decrease of inflammation in different tissues in obese mice treated with HMH2 and HMH3 and the improvement of glucose metabolism may be explained by the synergy between peptides, PUFA, and astaxanthin.

The role of the gut microbiota is now recognized as a key determinant in the development of metabolic disorders associated with obesity and diet is clearly an important player influencing its composition [31]. The LEfSe analysis revealed a significant increase in the relative abundance of *Lactobacillus* following HMH2 treatment compared to the HFHS control group, while a decrease was reported in comparison to the chow diet. Therefore, HMH2 appears to protect against the population reduction of *Lactobacillus* brought on by the HFHS diet. The present study is the first to show a modulation of the gut microbiota population following treatment with a herring milt hydrolysate product. Several *Lactobacillus* species have been used as probiotic strains and have shown beneficial effects, such as body weight and fat tissue reduction, as well as an improved insulin resistance and dyslipidemia [51,52,53,54]. For instance, *L. reuteri* limits obesity development in rats and increases glucose transporter 4 (GLUT4) gene expression in white adipose tissue WAT, indicating a potential improvement of glucose metabolism [51]. Another strain—*L. rhamnosus*—enhances glucose tolerance in mice fed a high-fat diet. The authors explained this improvement by a decrease of gluconeogenesis (suppression of G6Pase and PEPCK expression) and an increase of glucose uptake in the skeletal muscle (GLUT4 mRNA expression) [55]. Nevertheless, the genus *Lactobacillus* comprises more than 90 species, which makes it difficult to discern the relevance of specific subspecies or strains in the control of metabolism within the gut microbiota.

Finally, RNA seq analysis of the liver revealed that treatment with HMH2 induced a 1.29-fold increase (0.37 log2 fold change) in Nuak2 transcript expression versus the HFHS diet control. Nuak2, also termed SNARK, has been identified as a mediator of insulin-independent glucose transport, specifically regulating exercise- and ischemia-stimulated glucose transport in the heart and contraction-stimulated glucose transport in skeletal muscle [56,57]. However, our understanding of SNARK’s impact on glucose metabolism is still quite limited. SNARK belongs to the 5′ AMP-activated protein kinase (AMPK) family, signifying that their involvement in glucose metabolism in the liver may be similar [58]. Indeed, AMPK activation allows the inhibition of hepatic glucogenesis [59]. On the other hand, HMH2 treatment also induced a 1.5 log2 fold decrease in the FOXQ1 transcript expression when compared to the HFHS diet control. It has been shown that FOXQ 1 expression reduction in mice increased hepatic glucogenesis [60]. Consequently, further studies are needed to clarify the molecular mechanism of HMH products in the liver and their roles in the reduction of the development of obesity-related metabolic disorders.

## 5. Conclusions

In this study, low doses of HMH products were evaluated for their potential health benefits in a model of diet-induced obesity in mice and cell lines. During the OGTT, early time point glycemia was improved by HMH2 and HMH3, without changes in weight gain and insulin secretion. While HMH2 and HMH3 decreased inflammation in macrophages, no changes of glucose uptake in muscle cells were reported, suggesting that an indirect effect, such as inflammation reduction in different tissues, may have contributed to the improvement of glucose metabolism. Moreover, protection against the disappearance of *Lactobacillus* in HFHS-fed mice by HMH2 may also have contributed to the improvement in glucose tolerance. Additionally, some modulations of gene expression in the liver, such as the upregulation of SNARK, may explain the effects of HMH2. Finally, the mixture of peptides, PUFA, and astaxanthin contained in these herring milt hydrolysates may be useful for improving metabolic health. Therefore, the use of fish by-products such as herring milt in the development of new nutraceutical products is a promising way to improve their economic value. However, further studies are needed to clarify the availability and molecular mechanisms of these products in humans.

## Figures and Tables

**Table 1:** Chemical composition of the herring milt hydrolysate (HMH) products.

	HMH1	HMH2	HMH3
Protein/peptide (%)	93.79	48.28	47.0
Lipids (%)	-	18.48	26.0
Nucleic acid (%)	7.33	27.30	7
Astaxanthin (ppm)	-	-	500

**Table 2:** Effect of the HMH treatments on body characteristics.

	Chow	HFHS	HMH1	HMH2	HMH3
Total weight gain (g)	2.71 ± 0.23 ***	8.18 ± 0.66	9.71 ± 0.66	7.83 ± 0.60	9.48 ± 0.33
Total energy intake (kcal)	552.41 ± 29.42	592.65 ± 10.76	635.93 ± 12.83 ^#^	608.37 ± 11.35	632.59 ± 11.48
Visceral fat pad (g)	0.93 ± 0.05 ***	2.75 ± 0.30	3.20 ± 0.23	2.83 ± 0.22	3.14 ± 0.15
Subcutaneous fat pad (g)	0.29 ± 0.01 ***	0.69 ± 0.06	0.78 ± 0.06	0.67 ± 0.05	0.73 ± 0.03
Brown adipose tissue (g)	0.074 ± 0.003 *	0.092 ± 0.006	0.099 ± 0.004	0.089 ± 0.005	0.103 ± 0.006
Liver (g)	1.05 ± 0.02	0.92 ± 0.08	1.03 ± 0.03	0.99 ± 0.03	1.00 ± 0.03
Liver triglycerides (mg/g of liver)	17.34 ± 0.9 ***	40.86 ± 4.59	48.10 ± 4.88	45.31 ± 7.46	41.58 ± 2.99
Liver cholesterol (mg/g of liver)	7.83 ± 0.23 **	11.91 ± 1.45	13.28 ± 1.05	13.97 ± 2.67	11.86 ± 0.75

Mean ± SEM, * *p* < 0.05 chow vs. HFHS, ** *p* < 0.01 chow vs. HFHS *** *p* < 0.001 chow vs. HFHS, and ^#^ *p* < 0.05 HFHF vs. HMH1. HFHS: high-fat high-sucrose, SEM: standard error of the mean.

## Supplementary Materials

The following are available online at https://www.mdpi.com/2072-6643/12/11/3235/s1, Figure S1: Pathways significantly enriched from the list of 1994 differentially transcripts in HFHS diet vs Chow diet, Figure S2: Effect of HFHS diet on the Lactobacillus population in the gut microbiota of C57Bl/6J male mice after 8 weeks of treatment, Table S1: Transcripts showing differential expression a trend in HMH supplementations vs HFHS diet comparisons.
