# Supplementary material for: Animal and Cellular Studies Demonstrate Some of the Beneficial Impacts of Herring Milt Hydrolysates on Obesity-Induced Glucose Intolerance and Inflammation
Source: Nutrients. 2020 Oct 22;12(11):3235. doi: 10.3390/nu12113235 (PMC7690616; doi:10.3390/nu12113235)
Supplement: Supplementary file 1 [file nutrients-12-03235-s001.pdf]

Supplementary Figure 1. Pathways significantly enriched from the list of 1994 differentially transcripts in HFHS diet vs Chow diet.

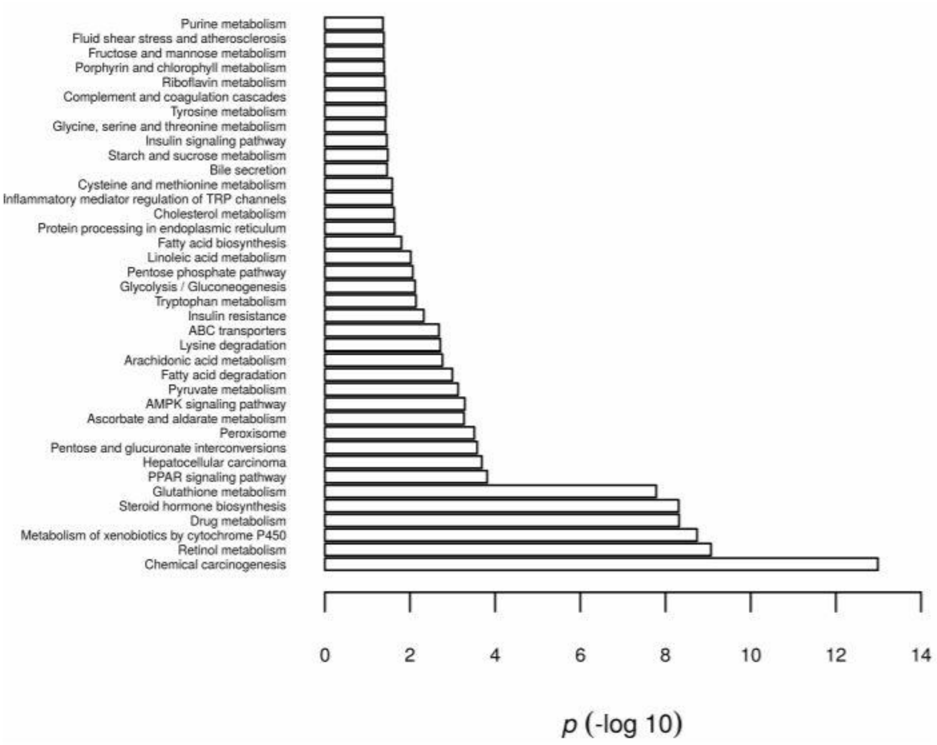

Supplementary Figure 2. Effect of HFHS diet on the Lactobacillus population in the gut microbiota of C57Bl/6J male mice after 8 weeks of treatment. ## $p < 0.01$.

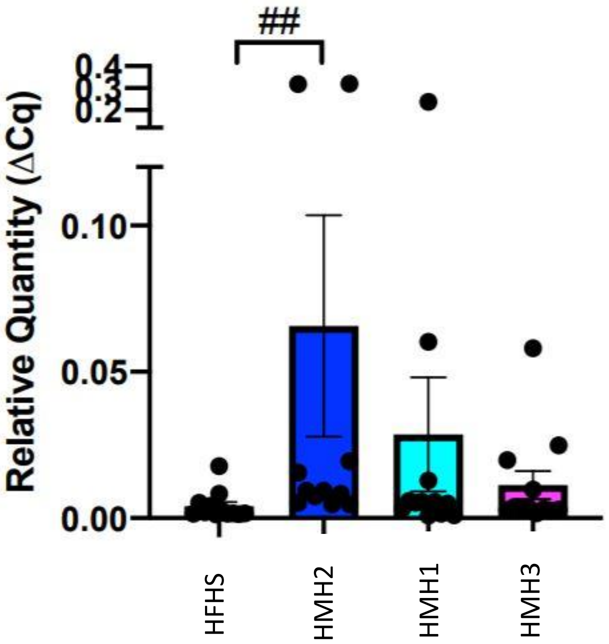

Supplementary Table 1. Transcripts showing differential expression a trend in HMH supplementations vs HFHS diet comparisons.

RefSeq mRNA	HMH1 logFC	HMH1 FDR p-value	HMH2 logFC	HMH2 FDR p-value	HMH3 logFC	HMH3 FDR p-value	Symbol	Gene names
NM_008484	7.325	0.0000127	7.11	0.0000338	0.03	1.000	Lamb3	laminin, beta 3
NM_009344	-0.283	1.000	-0.99	0.00593	-0.09	1.000	Phlda1	pleckstrin homology like domain, family A, member 1
NM_008239	-0.581	1.000	-1.50	0.00593	-0.31	1.000	Foxq1	forkhead box Q1
NM_008390	0.030	1.000	0.75	0.00593	0.47	1.000	Irf1	interferon regulatory factor 1
NM_028778	0.172	1.000	0.37	0.00998	0.30	0.496	Nuak2	NUAK family, SNF1-like kinase, 2
NR_028360	-0.292	1.000	-0.56	0.0509	-0.14	1.000	1700018L02Rik	RIKEN cDNA 1700018L02 gene
NR_045284	-0.389	1.000	-0.75	0.0509	-0.22	1.000	Gm16551	predicted gene 16551
NM_001177352	-0.667	1.000	-1.55	0.0509	-0.37	1.000	Myc	myelocytomatosis oncogene
NM_010849	-0.667	1.000	-1.55	0.0509	-0.37	1.000	Myc	myelocytomatosis oncogene
NM_008599	0.031	1.000	1.58	0.0509	0.27	1.000	Cxcl9	chemokine (C-X-C motif) ligand 9
NR_028384	-0.728	1.000	-1.16	0.0629	-0.37	1.000	4930528A17Rik	RIKEN cDNA 4930528A17 gene
NM_001195632	0.073	1.000	0.30	0.0629	0.12	1.000	Arhgap32	Rho GTPase activating protein 32
NM_001256184	0.250	1.000	0.62	0.0629	0.13	1.000	Gm5108	predicted gene 5108
NM_008330	0.124	1.000	0.87	0.0629	0.37	1.000	Ifi47	interferon gamma inducible protein 47
NM_021334	0.433	1.000	1.14	0.0629	0.38	1.000	Itgax	integrin alpha X
NM_021274	0.038	1.000	1.26	0.0629	0.35	1.000	Cxcl10	chemokine (C-X-C motif) ligand 10
NM_194336	0.066	1.000	1.23	0.0917	0.47	1.000	Gbp6	guanylate binding protein 6
NM_001355757	0.121	1.000	0.58	0.0930	0.26	1.000	Irgm1	immunity-related GTPase family M member 1
NM_001039160	0.159	1.000	1.19	0.0930	0.47	1.000	Gvin1	GTPase, very large interferon inducible 1
NM_001291067	-0.511	1.000	-0.93	0.0985	-0.49	1.000	Foxa2	forkhead box A2
NM_008509	0.065	1.000	0.32	0.0998	0.07	1.000	Lpl	lipoprotein lipase
NM_010260	0.090	1.000	1.44	0.0998	0.43	1.000	Gbp2	guanylate binding protein 2

Abbreviations: logFC, log2 fold change; FDR p-value, false discovery rate-corrected p-value; HFHS, High fat high sugar.
